# On Hæmorrhage from Waxy or Amyloid Degeneration

**Published:** 1868-06

**Authors:** T. Grainger Stewart


On Hæmorrhage from Waxy or Amyloid Degeneration.—By T. Grainger Stewart, M. D., F. R. S. E., etc. Dr. Stewart thinks that the following conclusions are warranted by the facts thus far observed in connection with the subject:

1. That hæmorrhage is not a very infrequent consequence of the waxy or amyloid degeneration of the vessels
2. That, next to the spleen, the intestinal tract is the most common seat of such hæmorrhage.
3. That the hæmorrhage occurs independently of any visible ulcerative process.
4. That it probably depends upon rupture of the capillaries of the affected parts.

5. That waxy or amyloid degeneration of the liver does not of itself suffice to produce hæmorrhage from the bowels.

6. That the hæmorrhage occurs in cases in which the liver is free from waxy degeneration.

7. That the occurrence of hæmorrhage increases the danger of the patient. But—

8. That sometimes it comes and goes for years without markedly depressing the vital powers —*British and Foreign Medico-Chir. Review, January*, 1868.

